# On the within-person effects of political attitudes on outgroup animosity: Evidence for Democratic-Republican asymmetry

**DOI:** 10.1371/journal.pone.0352103

**Published:** 2026-09-08

**Authors:** Yaacov Schul, Yoav Ganzach, Asya Pazy

**Affiliations:** 1 The Hebrew University, Jerusalem, Israel; 2 Tel Aviv University, Tel Aviv, Israel; University of Malta, MALTA

## Abstract

On the basis of a within-person model of the relationship between political identity and outgroup dislike, Brandt and Vallabha (2025) suggested that changes in the strength of party identity, but not changes in the direction of this identity, are correlated with political animosity. These results are particularly interesting since they are consistent with the symmetric view of political ideology, which argues that partisans are similarly intolerant toward their political opponents. In this paper, we highlight conceptual and methodological issues that cast doubt on the validity of BV’s conclusions. We also propose an alternative model and apply it to BV’s data, alongside data from a second large dataset. Our findings indicate that Democratic and Republican respondents differ in the effect of identity strength on animosity, a result which is consistent with the asymmetric, rather than the symmetric, view. We conclude by discussing the methodological and conceptual insights gleaned from these findings.

## Introduction

The conventional view on the relationship between political ideology and intolerance suggests that political conservatism is associated with higher levels of outgroup intolerance (e.g., [1, 2]; see review in [3]). This perspective has recently been challenged by scholars advocating the “symmetric” view, which proposes that liberals/Democrats and conservatives/Republicans exhibit similar levels of intoleranceoutgroup [3–5]. Utilizing *within-person* design, the research described below investigates whether Democrats and Republicans exhibit comparable levels of intolerance sensitivity toward their respective outgroups.

Past research examining the symmetry/asymmetry question typically employs *between-persons* designs, in which respondents are classified as Democrats or Republicans based on their political affiliation (or as liberals/conservatives based on their ideology), and their animosity toward an outgroup is then assessed. However, because the between-persons designs are mute with respect to within-person dynamics in general [6], and temporal changes in one’s sense of party affiliation in particular, the utilization of such designs might be problematic. This limitation is particularly significant since the between-persons analyses do not necessarily generalize to within-person designs [7,8]. This reasoning was recently embraced by Brandt and Vallabha (1, BV, hereafter). Using *completely within-person analyses*, they examined the relationship between components of party identity and outgroup animosity. They concluded that changes in the *strength* of party identity, but not changes in the *direction* of this identity, are associated with variations in political animosity expressed toward outgroups. These results are particularly noteworthy since they are consistent with the symmetric view of political ideology, which posits that partisans on both sides exhibit comparable levels of intolerance

In this paper, we revisit the completely within model, discussing the usefulness of applying within-person models instead of between-persons models to predict outgroup animosity. We have two goals in mind.

The first, is more methodological in nature. We illustrate the potential difficulties of within-person models by critically evaluating the completely within-person model of BV [9]. Specifically, we identify conceptual and methodological issues in their fully within-person approach that call their conclusions into question. In response to these difficulties, we propose an alternative within/between model for studying the relationship between party identity and outgroup animosity. While building the model, we discuss the cogency of the predictors employed by BV in a within-person design. The alternative model is used to reanalyze BV’s data together with a second large dataset (VOTER, see detailed below).

These analyses allow us to pursue our second goal, namely, examine the symmetry/asymmetry between Democratic and Republican respondents with respect to outgroup animosity. In a nutshell, using a within/between statistical design, our analyses demonstrate that (i) outgroup animosity increases with the distance between one’s political identity and the outgroup; and (ii) Democrats exhibit reduced sensitivity to the distance from their outgroups compared to Republicans. The latter finding is consistent with the asymmetric, rather than the symmetric view.

### The between-persons model of party identity and strength

The conventional approach to studying the relationship between political identity and outcomes such as outgroup attitudes employs a between-persons design [10–12]. In this approach, it is common to represent political identity using two conceptually orthogonal derivative variables: a binary party identity (e.g., Democratic vs. Republican) and an index of identity strength. For example, when identity is measured on a 7-pt party-affiliation scale, ranging from strong Democrat [9] to strong Republican [6] PID, the binary identity variable, indicates whether the participant is Democrat (having a value smaller than 4 on the 1–7 scale) or Republican (a value greater than 4). Strength is computed as the unsigned value of the discrepancy of identity from the midscale [3].

This conceptualization is particularly appropriate in those surveys that measure party identity using a two-question format involving inquiries about binary party identification and the strength of that identification (cf., [13]) (To illustrate, see the Method section for the elicitation procedure in the VOTER survey). The two derivative indicators are then used in a linear fashion to model outcomes, and in particular attitudes toward the outgroup, in line with equation 1 below (This model is the most basic model to estimate the effects of party identity and strength on dislike, and it estimates only their main effect). In this model PIDi is the binary party identity of respondent i (i.e., Democratic or Republican), STRi is a strength indicator, defined as the unsigned discrepancy from the scale’s midpoint, and ATTi, indicates the attitude expressed toward the outgroup. To examine outgroup animosity, the attitude response is reversed.


$$\begin{array}{c} {\text{ATT}}_{\text{i}}=\;\beta \text{0}\;+\;\beta \text{1}*{\text{PID}}_{\text{i}}+\;\beta \text{2}\;*\;{\text{STR}}_{\text{i}} \end{array}$$ (1)

### The completely within-person model

Within-person analyses capitalize on the dynamics within a person. The focus is on transient changes across different times of measurement. To control for individual differences, the predictor variables are mean-centered [14]. Translating the between-persons design, yet following the logic of the within-person design, BV propose to partition the party affiliation rating in each wave into two component variables: Within-person transient changes in the direction of the political identity and within-person transient changes in the strength of the identity. Consistent with the practice in the within-person analyses, these two derivative variables are centered within respondent. We designate the mean-centered transient changes in political identity as CPID. Similarly, we designate the transient wave score for strength as CSTR. Appendix 1 provides detailed description how these variables are derived. The dependent variable in BV’s model is the attitude expressed toward the outgroup at each specific wave. Using these definitions, BV propose the completely within-person model expressed by equation 2 where i refers to respondents, and k denotes the wave:


$$\begin{array}{c} {\text{ATT}}_{\text{ik}}\;=\;\gamma \text{0}\;+\;{\gamma \text{1}}_{\text{i}}*{\text{CPID}}_{\text{ik}}\;+\;{\gamma \text{2}}_{\text{i}}*{\text{CSTR}}_{\text{ik}} \end{array}$$ (2)

Because, at a first glance, the between-persons (equation 1) and completely within-person (equation 2) seem similar, the conceptual reasonableness of the within-model has not been questioned. BV estimated this model and found (i) a statistically significant γ2 coefficient, suggesting that outgroup animosity increases as the strength of party affiliation increases within a person; and (ii) a non-significant γ1 effect, indicating that within-respondent changes of party identity in the Democratic direction have similar effects as within-respondent changes in identity in the Republican direction, in line of the symmetry hypothesis. However, as we demonstrate below, the translation of a between-persons design to a within-person design creates several theoretical and empirical problems that cast serious doubts on the validity of BV’s conclusions.

Specifically, we identify two primary issues with the BV model, each pertaining to a distinct subpopulation of respondents. The first issue involves respondents we term “non-crossers.” These individuals did not cross the party line (i.e., the scale midpoint) in any of the waves when reporting their party identity. That is, they consistently reported a Democratic identity or a Republican identity across waves. Non-crossers represent the vast majority, exceeding 95%, of the relevant samples (see below). The observed stability of partisan identification among respondents is consistent with accounts that conceptualize party identification as a social identity, rather than as a short-term evaluation of political performance [15,16]. The second issue pertains to a small subpopulation of “crossers”, those who reported a Democratic identity in some waves and a Republican identity in others. Although crossers constitute a small subsample (see below), their presence raises a significant theoretical concern regarding the validity of the indicator of identity strength.

In the next two sections, we examine these issues in detail. Subsequently, we introduce an alternative model that is deemed more suitable given the complexities inherent in the completely-within model. This alternative model is employed to test the hypothesis about symmetry between Democrats and Republicans.

### The challenge posed by non-crosser respondents?

Examination of the model depicted in equation (2) reveals that the two predictors, CPID and CSTR, are perfectly correlated for non-crosser respondents. Under perfect collinearity, (i) the model cannot separately identify the effects of the two predictors, and (ii) infinitely many combinations of coefficients yield identical model fit. To illustrate one consequence of such a perfect collinearity, consider the two hypothetical respondents described in Table 1. Focusing on wave 1 of the Democrat (R1) and wave 4 of the Republican (R2), both have a CPID score of −1, indicating that both respondents shift their identity in the Democratic direction. Note that R1 is a Democrat in all 4 waves and R2 is a Republican in all 4 waves. Therefore, with respect to one’s partisan identity, the meaning of a transient identity, the “Democratic shift” in this example, is unclear. As we show below, apparent changes in the identity component, in fact, reflect changes in identity strength.

**Table 1:** Computation of the predictors used by BV in their within-person model.

		Changed-identity score			Changed-strength score		
Respondent	wave	WPID	MPID	CPID	WSTR	MSTR	CSTR
			MEAN(WPID)	(WPID-MPID)	|WPID-4|	MEAN(WSTR)	(WSTR-MSTR)
R1 (Dem)	1	1	2	−1	3	2	1
R1 (Dem)	2	2	2	0	2	2	0
R1 (Dem)	3	2	2	0	2	2	0
R1 (Dem)	4	3	2	1	1	2	−1
R2 (Rep)	1	7	6	1	3	2	1
R2 (Rep)	2	6	6	0	2	2	0
R2 (Rep)	3	6	6	0	2	2	0
R2 (Rep)	4	5	6	−1	1	2	−1
R3 (Rep)	1	3	5	−2	1	1.5	−0.5
R3 (Rep)	2	5	5	0	1	1.5	−0.5
R3 (Rep)	3	6	5	1	2	1.5	−0.5
R3 (Rep)	4	6	5	1	2	1.5	−0.5

Notes: *WPID* refers to wave-level reported party identity. *CPID* indicates the BV’s mean-centered transient changed in identity predictor and *CSTR* denotes BV’s mean-centered transient changed in-strength predictor. Participant R3 is considered a *Crosser*, reporting a Democratic identity in wave 3 and Republican identities in waves 2, 3 and 4. Respondents R1 and R2 are considered non-crossers, as they do not cross the party line in reporting their identity.

To see why, consider the Democrat (R1 in Table 1). A downward change (as in wave 1) means moving away from the scale’s mid-point, that is, having a stronger identity (Party identity scale goes from 1 (strong Democrat) to 7 (strong Republican). Therefore, a downward change means moving toward the Democratic end of the scale); In contrast, an upward change, that is, moving toward the Republican end of the scale (as in wave 4), means getting closer to the mid-point, that is, having weaker identity. Thus, for a Democrat, the two components, transient change in identity (CPID) and transient change in strength (CSTR) mirror each other, they exhibit a perfect negative correlation.

For a Republican (see R2 in Table 1), the correlation between CPID and CSTR is positive. A downward change in the party identity (movement toward the Democratic end) means movement toward the midpoint, and therefore, a reduction in identity strength; an upward change (movement toward the Republican end) means increase in identity strength. For non-crosser Republican respondents, CPID and CSTR exhibit a perfect positive correlation.

Importantly, these correlations are not contingent on the specific values of wave party identity presented in Table 1. They occur for all respondents who do not cross the party line when expressing their party identity. Table 2 reveals that more than 95% of the respondents who exhibit identity change are non-crossers. Accordingly, for vast majority of the respondents, the CPID-CSTR correlations are either −1 (Democrats) or +1 ((Republican), meaning that transient changes in identity are perfectly aligned with transient changes in strength. This raises the question: Given this perfect correlation within the large majority of individuals, how is it that BV found that changes in strength and political animosity are significantly correlated, while observing no such associations with changes in political identity? We return to this paradox in detail in the Discussion section.

**Table 2:** Classification of respondents in the two datasets.

	VOTER Dataset		BV Dataset
	Only waves with attitude measurement	All waves	All waves
N of waves	5	9	26
*Irrelevant* Rs (a single WPID or all missing)	5112	2486	22
*Uniform* Rs (identical WPID in all non-missing waves)	5452	5567	214
*Variable* Rs (variable pattern of WPID)	1953	4464	316
Total N	12517		552
% irrelevant Rs (out of total sample)	41%	20%	4%
% variable Rs (out of uniform and variable Rs)	26%	45%	60%
Count of Rs who crossed party line (Crossers)	192	582	20
%crossers (out of uniform and variable Rs)	2.59%	5.80%	3.77%

Notes: WPID indicates the wave-level reported party identity. In the BV dataset, party identity (WPID) and attitude responses were collected in each wave. In the VOTER dataset WPID responses were recorded in 9 waves, but attitude measures were included only in 5 of them.

### The challenge posed by crosser respondents

There is, however, a small subpopulation of respondents, whom we call “crossers”, who report a Democratic identity in some waves and a Republican identity in others. For these respondents, unlike the noncrossers, the mean within-respondent correlation between CPID and CSTR varies between individuals. To illustrate, in the BV sample, the correlation between the two measures is 0.046 (SD = 0.570).

The presence of crosses raises a question concerning whether the strength of party identity is the appropriate construct for predicting outgroup attitude. BV’s model conceptualizes strength in a manner akin to the between-persons design, viewing it as the size of the deviation from the scale’s midpoint (see Appendix 1). However, in the context of a within-person design for the relationship with outgroup animosity, there are reasons to believe that what drives outgroup attitudes is the *distance* from the outgroup, rather than the deviation from the midpoint. Specifically, a large body of research is consistent with the similarity-attraction principle, which posits that greater dissimilarity leads to reduced attraction [17–22]; see [23], for an extension to the domain of Political Psychology). In the context of a 7-point political identity scale, anchored by 1 = DEM and 7 = REP, applying this principle suggests that a Democrat’s attitudes toward the Republican Party should be more positive when their wave party identity (WPID) is 5 than when it is 3, as they are closer to the Republican Party when WPID is 5. Similarly, a Republican’s attitudes toward the Democratic Party should be more negative when WPID = 5 compared to WPID = 3, as they are closer to the Democratic Party at WPID = 3. This perspective suggests that attitudes are influenced by the distance (dissimilarity) from the target party, rather than the strength score – the unsigned discrepancy from the midpoint (Operationally, when party identity is assessed using a 7-point scale, the distance a Democrat from the Republican Party might be estimated by 7-WPID, and the distance of a Republican from the Democratic Party might be estimated by WPID-1).

The theoretical predictions of the “distance” conceptualization are at odds with the theoretical within-person model involving strength. To illustrate, consider the case of a crosser, R3, in Table 1. R3, expresses weak Democratic and Republican identities in different waves: WPID = 3 in wave 1 and WPID = 5 in wave 2. Since the wave strength score is the unsigned difference from 4, the two strength scores of R3 in waves 1 and 2 are identical. Because R3’s outgroup is the Democratic party, the theoretical prediction of BV’s “strength” model implies that animosity toward the Democratic party would be unaffected by whether WPID = 3 or WPID = 5 —an implausible prediction.

Before proceeding to describe an alternative model, let us make two caveats. First, for the majority of the individuals, that is, for all non-crossers, the distance conceptualization and BV’s strength conceptualization make the same predictions regarding animosity. This equivalence implies that the distance and strength models differ only for crosses, namely, when respondent’s identity ratings cross the mid-point (e.g., 3 and 5 on a 7-point scale). Second, it is important to note that the theoretical difficulties associated with the strength model occur only in the within-person models. In between-persons designs each respondent expresses only a single party identity. Therefore, a person with a party identity of 3 is a Democrat while a person with party identity of 5 is a Republican. As a result, when the effect of 3 and 5 on animosity are compared, they refer to different outgroups. We further elaborate this point in the Discussion Section.

### An alternative within/between model

Our analysis of the completely within-person model has identified two difficulties. First, the model employs two within-person predictor variables –transient changed identity and transient changed strength -- which correlate perfectly within a person for the vast majority of respondents (i.e., for all the non-crossers). Second, based on past research, we reasoned that attitudes toward the outgroup are influenced by the distance (dissimilarity) from the target party, rather than by the strength of identity – the deviation from the scale midpoint. The alternative model we introduce below is informed by these difficulties.

Consider, first, the reduced main-effect-only model, which is expressed in equation 3r, where i designates respondent and k designates wave.


$$\begin{array}{c} {\text{ATT}}_{\text{ik}}\;=\;\delta \text{0}\;+\;\delta \text{1}*{\text{PID}}_{\text{i}}\;+\;\delta \text{2}*{\text{CDISTANCE}}_{\text{ik}} \end{array}$$ (3r)

In this model, CDISTANCE represents the transient change in the psychological distance between the identity of the respondent and the outgroup. It is a centered within-person predictor, calculated as the difference between the distance in wave k and the mean distance across all waves, computed within each respondent. The other predictor in the model is the binary party identity (PID). It is considered a level-2 indicator, namely a personal characteristic that does not vary between waves. The necessity of modeling PID as a between-persons predictor reflects the constraint that for nearly everyone, transient change in party identity means transient change in strength/distance and vice versa. Accordingly, only one of the two within-person predictors could be retained as a within-person predictor. Because for 95% of the respondents, party identity is stable across waves (i.e., respondents do not switch parties in different waves), our model treats the binary party identity (Democratic vs. Republican, see below) as a between-persons construct. It represents the respondent’s long-term party identity, which is estimated by the average party identity across all waves, Respondents with an average identity below the midpoint were classified as Democrats (-1), while those above the midpoint were classified as Republicans (+1).

The main-effect model in equation (3r) is similar in structure to the fully between-persons model (equation 1) and the fully within-person model (equation 2). However, this model allows testing of the question of differences between Democrats and Republicans regarding outgroup animosity (the symmetry/asymmetry question) in a very limited way. Specifically, it permits testing only whether Republicans and Democrats, as two groups, vary in their animosity toward their political opponents. Yet, there is another important sense of the symmetry/asymmetry question: the sensitivity of Republicans and Democrats to the distance from their political opponents. Testing this question requires the main-effect+interaction model specified in equation 3 below (and see Appendix 2 for a formal development).


$$\begin{array}{c} {\text{ATT}}_{\text{ik}}\;=\;\delta \text{0}\;+\;\delta \text{1}*{\text{PID}}_{\text{i}}\;+\;\delta \text{2}*{\text{CDISTANCE}}_{\text{ik}}\;+\;\delta \text{3}*\;{\text{PID}}_{\text{i}}*{\text{CDISTANCE}}_{\text{ik}} \end{array}$$ (3)

Based on model expressed in equation 3 the question of symmetry/asymmetry is tested by examining two parameters: (i) the overall difference between Democrats and Republicans, which is indicated by δ1, and (ii) the Democrats/Republican similarity in the magnitude of association between changes in distance and changes in the attitudes expressed toward the outgroup, which is indicated by the magnitude of δ3.

## Method

Our analyses are conducted using two datasets, termed BV and VOTER, which were collected around the same time. These studies employ different questions to assess party identity and party attitude.

### The BV dataset

As detailed in Brandt and Vallabha [9], this dataset is based on the 26-wave Yearlong Longitudinal Study conducted between 2019 and 2020, which involved regular biweekly reports from participants between May 2019 and April 2020. The dataset comprises responses from 552 U.S. American participants recruited through Prolific. Each wave of the study includes self-reported party identity as well as attitude ratings (see below). Additional information can be found at https://osf.io/sefg9/.

### The VOTER dataset

The Democracy Fund Voter Study Group is a research project aimed at providing insights into the evolving perspectives of American voters (see https://www.voterstudygroup.org/). The project’s goal is to assist policymakers and thought leaders in better understanding and responding to the views of American voters. This dataset comprises of 9 waves (2011, 2012, 2016, 2017*, 2018, January 2019*, November 2019*, September 2020*, November 2020*). Party identity ratings were collected in all 9 waves, while ratings of party attitudes were collected in the 5 starred waves. The VOTER dataset and its documentation can be accessed at https://www.voterstudygroup.org/downloads/voter-survey?key=800521.

### Respondents

Respondents in each dataset were classified into three types: *irrelevant* respondents (those who are irrelevant for analysis of change because they have fewer than two waves with valid WPID scores), *uniform* respondents (those who are irrelevant for analysis of change because their WPID responses were identical in all waves), and *variable* respondents, whose data can be analyzed to test questions about the effect of identity change. Classification of respondents in the two datasets appears in Table 2.

The bottom rows in Table 2 present the count and rate of crossers – those respondents who expressed a Democratic identity in one wave and a Republican identity in another wave. It is clear that crossing is rare. Across both datasets, the vast majority of *variable* respondents did not switch party identities.

## Measures

### Party identity (BV)

Respondents were asked, “Do you think of yourself as a Republican, a Democrat, an Independent, or haven’t you thought much about this?” Responses were made on a 7-point scale (Strongly Democrat, Democrat, Independent-lean Democrat, Independent, Independent-lean Republican, Republican, Strongly Republican). Non-substantive answers (“Don’t Know” and “I haven’t thought much about it”) were considered missing in our analyses.

### Party identity (VOTER)

Respondents were first asked: “Generally speaking, do you think of yourself as a...?” with the response options being Democrat, Republican, Independent, Other, and “Not sure”. Those answering Democrat or Republican were further asked, “Would you call yourself a strong [Democrat/Republican] or a not very strong [Democrat/Republican]? Those saying Independent, Other, or “Not sure” were further asked, “Do you think of yourself as closer to the Democratic Party or the Republican Party? Answers to the two questions were combined to a 7-point scale ranging from Strong Democrat [9] to Strong Republican [6]. “Not sure” was considered missing in our analyses. Note that party identity is assessed using a single-item format in BV and a 2-question format in VOTER. Thus, our results generalize across different measurement formats.

### Party Attitude (BV)

Respondents were asked, “How willing would you be to be friends with people from the following groups? (Republicans/Democrats). Responses were given on a 7-point scale anchored by ”I absolutely would not” [9] and “I absolutely would” [6]. “Don’t know” and “I haven’t thought much about it” were considered missing values in our analyses.

### Party Attitude (VOTER)

Respondents were asked, “We’d like to get your feelings toward some groups who are in the news these days. Ratings between 50 degrees and 100 degrees mean that you feel favorable and warm toward the group. Ratings between 0 degrees and 50 degrees mean that you don’t feel favorable toward the group and that you don’t care too much for that group. You would rate the group at the 50 degree mark if you don’t feel particularly warm or cold toward the group. If we come to a group who you don’t recognize, you don’t need to rate that group. Click on the thermometer to give a rating to [Democrats/Republicans]. The two datasets employ different measures of attitudes toward the opposing party, allowing for generalization across measurement formats. To allow comparison between the attitude responses in the two datasets, they were rescaled to 0–1 range.

Our main analyses focus on attitudes toward the opponent party. We follow the practice of BV and determine the opponent party based on the mean party identity of the respondent.

**Preregistration**. The study was not preregistered.

## Results

To estimate the statistical model expressed in equation 3 we employed SAS PROC MIXED to conduct a two-level mixed-model analyses predicting the variations in the attitude toward the outgroup. Specifically, the attitude was predicted from the changed-distance scores (a level-1 predictor), the Dem/Rep party identification indicator (a level-2 predictor), and the cross-level interaction between changed-distance score and Dem/Rep classification. Additionally, we included a set of dummy variables for the waves to account for systematic between-waves variance in the analysis.

**Fig 1:**
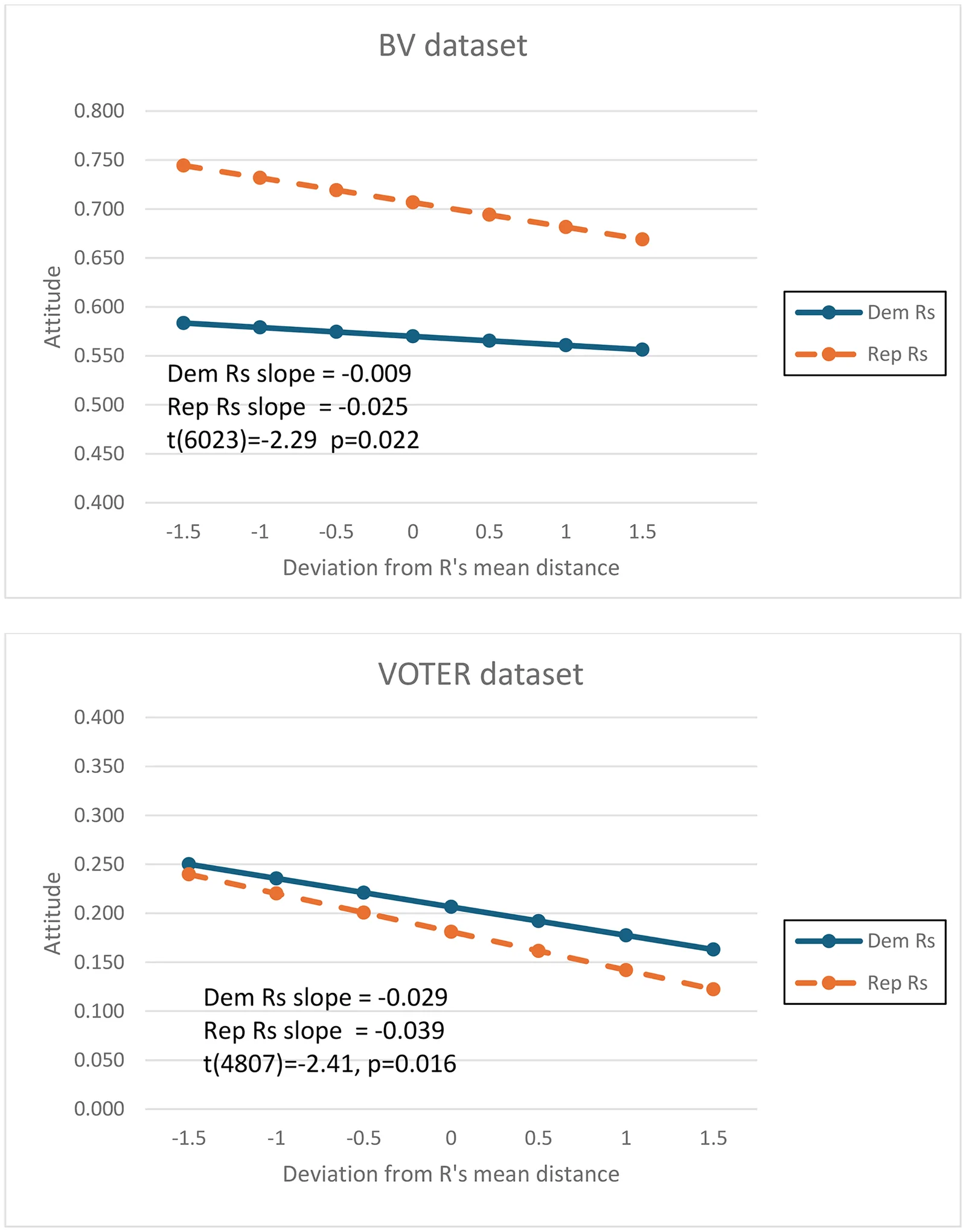
Predicted mean attitude toward the opponent party as a function of the wave changed distance score and the long-term party identity. Notes: Predicted attitudes toward the opponent party are based on the statistical model presented in the text. Attitudes are scaled to the 0-1 range. Negative/positive deviations (X axis) indicate being closer/farther to/from the opponent party relative to the respondent's mean distance. Negative slopes indicate that the farther away one is from the opponent party, the less positive the party becomes.

Table 3 displays the results of the mixed-model analyses, and Fig 1 illustrates the predicted means based on the model’s coefficients. The effect of distance is substantial in both datasets, with coefficients of −0.017 (95% CI: −0.024, −0.01) in the BV dataset and −0.034 (−0.038, −0.029) in the VOTER dataset. The negative sign indicates that as the distance from the opponent party *increases*, the favorability of attitudes toward that party *decreases*.

**Table 3:** Results of the mixed-model analyses predicting attitude toward the opposing party from long-term party identity and the changes in distance to the opponent party.

BV dataset					
**Effect**	**Estimate**	**Standard**	**DF**	**t Value**	**Pr > |t|**
		**Error**			
Intercept	0.638	0.016	313	38.82	<.0001
Dem/Rep	0.068	0.015	313	4.46	<.0001
Changed distance	−0.017	0.004	6023	−4.87	<.0001
Dem/Rep*distance	−0.008	0.004	6023	−2.29	0.022
**VOTER dataset**					
**Effect**	**Estimate**	**Standard**	**DF**	**t Value**	**Pr > |t|**
		**Error**			
Intercept	0.194	0.006	1886	32.42	<.0001
Dem/Rep	−0.013	0.005	1886	−2.69	0.0071
Changed distance	−0.034	0.002	4807	−16.12	<.0001
Dem/Rep*distance	−0.005	0.002	4807	−2.41	0.0158

Notes: Sample sizes: BV = 313, VOTER = 1886. Attitude is rescaled to 0–1 range. Long-term party identity (Dem/Rep) is coded Dem = −1, Rep=+1. Changed-distance is a wave-level predictor that represent the distance to the opponent party in a particular wave relative to the respondent’s mean distance. The statistical model included a set of dummy variables to remove between-waves variability (not shown).

Importantly, as shown in Fig 1, the negative slope differs between Democrats and Republicans: Republicans exhibit a steeper slope than Democrats. This asymmetry is statistically significant, as indicated by a significant interaction, with coefficients of −0.008 (−0.015, −0.001) in the BV dataset and −0.005 (−0.009, −0.001) in the VOTER dataset. This pattern suggests that Republicans reacted more strongly to changes from their mean. In particular, when they express identities that are more/less distant from the opponent party, they express an increasing/decreasing level of animosity toward that party. To aid interpretation of the coefficients, it is useful to translate them back to the original attitude scale. To illustrate, consider the top panel of Fig 1, which presents the analysis of the BV data. It shows that when a Republican or Democrat becomes more extreme by shifting one point on the party-identity scale, their attitude toward the out-party changes by 0.025 and 0.009 units, respectively, on the transformed 0–1 scale. These correspond to approximately 0.175 and 0.063 units on the original 7-point scale.

Finally, let us point an inconsistency between the two datasets regarding the main effect of the Dem/Rep party classification. In the BV dataset, Democrats, on average, show reduced liking of their opponent party compared to Republicans, with an effect of 0.068 (0.038, 0.098). In contrast, in the VOTER dataset, Democrats show, on average, more liking toward their opponent party, with an effect of −0.013 (−0.022, −0.0035). Although the presence of any main-effect due to party classification is inconsistent with the hypothesis of Democrat/Republican symmetry, the inconsistent pattern in the two datasets begs the question why? We note that the two surveys assessed attitudes differently: the BV survey focused on the willingness to befriend members from the opposing party, and the VOTER survey utilized “thermometer” ratings, an indicator of affective reaction. We speculate that there are differences in the way Democrats and Republicans react to these questions (see the section titled “The symmetry question” in the General Discussion). Main effects due to party classification, therefore, might include such differences. Importantly, within-person designs utilize change scores. Therefore, they control for individual differences. Accordingly, the interaction effect, may indicate that regardless of the scales used to measure attitudes, Republicans react more strongly to changes in party identity. Thus, the pattern of inconsistent main effects, and consistent interactions may in fact highlight the usefulness of within-person designs.

## Discussion

Within-person designs offer a valuable methodological framework for examining changes within individuals. These designs compare each individual’s scores to their own average, thereby reducing error variance stemming from individual differences and controlling for stable individual characteristics that might otherwise confound results. Such designs are particularly well-suited for studying dynamic processes. Brandt and Vallabha [9] advocate for a completely within-person model to explore the association between individuals’ party identity and their animosity toward the opposing party. While we applaud their general approach, we believe that in the identity/animosity context a completely within-person model in general, and the specific model proposed by BV in particular, present several theoretical and methodological challenges. Below we discuss these challenges in turn.

### The question of generalization

The efficacy of within-person designs relies on the presence of variability in the predictor variables – the ratings of party identity. However, as shown in Table 2, a significant portion of respondents in both the BV and VOTER samples reported identical party identities throughout the study period. For these *uniform* respondents, there is no variation in the direction and strength of party identity, and consequently, within-person designs cannot handle this subgroup. This raises questions about our understanding of the relationship between identity and animosity in this subgroup, and prompts consideration of the generalizability of findings based on *variable* respondents to *uniform* respondents. Moreover, among those who expressed variable identities, most (over 95%) identified with a single party in all the waves. For these respondents, it seems more appropriate to treat variation in the binary party identity as a between-persons (level-2) factor. Addressing these questions necessitates further research that inherently combines within- and between-persons designs.

### The question of the perfect correlation between the two component scores

A second challenge to the completely within-person model BV proposed arises from its conceptualization of the party-identity continuum. Similar to the between-persons approaches, BV consider party identity as comprising two components: transient change in identity (CPID) and transient change in strength (CSTR). However, we showed that the two components exhibit a perfect correspondence for the majority of respondents. Given this perfect correlation within individuals, how is it that BV found that changes in strength predictive of variability in political animosity, while observing no such associations with changes in political identity?

We believe that two factors account for this puzzle. The first reflects the opposite signs of the correlations between CPID and CSTR for Democrats and Republicans. Specifically, for Democrats, animosity toward the opposing party increases when identity shifts toward the Democratic end of the scale, whereas for Republicans, such a shift is associated with decreased animosity. Conversely, for Democrats, animosity decreases when identity shifts toward the Republican end of the scale, but for Republicans, such a shift is linked to increased animosity. Taken together, when a sample of respondents contains both Democrats and Republicans, these opposing tendencies offset each other, leading to the conclusion that the overall effect of change in identity (CPID) on animosity is null, as observed by Brandt and Vallabha [9].

Second, the perfect correlation between the two transient change scores is not universal; it only occurs when respondents are non-crossers. The presence of crossers obscures the perfect correspondence between changed identity and changed strength, as seen in the vast majority of non-crossers, thus allowing the statistical analysis to converge. Consequently, despite the perfect correlation between identity change and change in strength for nearly all respondents, Brandt and Vallabha concluded that changes in strength are linked to variations in animosity, while changes in identity are not associated with animosity.

### The challenge of the defining the outgroup

The use of the distance indicator underscores the importance of identifying the opponent party. BV’s model, and consequently our own model, utilize the long-term binary identity (estimated by the mean party identity) to determine that party. However, this is not a necessary theoretical stance. An argument could be made for defining the opponent party variably, based on the respondent’s party identity in a specific wave. According to this approach, a change in wave identity is considered complete, rendering the history of party affiliation irrelevant. However, given the relative stability of party identities, noting that very few respondents crossed the party line while expressing their political identity, we consider this theoretical possibility unlikely. Nonetheless, it warrants investigation in future research.

### The symmetry question

Brandt and Vallabha [9] argued that changes in party identity do not affect animosity. Our analyses contend that such a claim is not warranted. Instead, we proposed to address a different question, which may be more central to theories of party identity: whether Democrats and Republicans are equally responsive to changes in the distance of their wave identity from their outgroup. The findings suggest that Republicans are more responsive than are Democrats; that is, their animosity toward the opponent party changes more drastically in response to shifts in their party identity. Practically, this implies that in times one reports extreme identities, outgroup animosity is more extreme among Republicans than among Democrats. This fits well with the traditional perspective on the psychological characteristics of prejudice [11,12,24], but conflicts with the symmetric school of prejudice [23]. We believe that Republicans may be more sensitive to extremity than Democrats because conservative psychology tends to place greater emphasis on social order, normative stability, and threat management [24–26]). Consequently, ideologically distant groups or persons tend to be seen by Republicans not simply as political opponents, but also as posing a more fundamental threat to the social and moral order. Further research—drawing on diverse methodologies and cultural contexts—is needed to more definitively assess the symmetry question.

## Conclusions

We conclude by emphasizing the complementary nature of within-person and between-persons designs. Each design is distinct and cannot replace the other, and theoretical constructs with similar labels may operate in dissimilar ways. Let us start by noting that although partisanship is largely stable, genuine changes may occur over a person’s lifespan [27]. Short-term fluctuations are constrained because partisan identities are reinforced by social sorting and media ecosystems [28]. Still, there is evidence that parties’ issue positions matter [29], although the issue effects are complex, depending among other things on the specific composition of the sample being studied, respondents’ political sophistication, as well as contextual conditions such as ethnic fractionalization or wealth inequality [30]. Therefore, in spite of the great amount of control a within-design offers, caution should be taken in generalizing from any specific sample (For example, as one reviewer noted, the BV dataset is based on a panel sample in which respondents completed biweekly surveys across 26 waves, implying a substantial level of commitment. This may indicate that respondents in the BV sample were comparatively more politically engaged, attentive to politics, and potentially more ideologically committed than respondents in the VOTER sample, where such intensive participation demands were less pronounced).

Accordingly, we propose that inferences drawn from both designs should be considered jointly, as each presents distinct challenges for generalization. For example, our finding of a more pronounced distance effect among Republicans relies specifically on respondents whose party identification varied over the study period. By contrast, respondents who maintained a stable party identity throughout the study (approximately half the sample) can only be analyzed using between-person designs, since there is no within-person variation to estimate. A comprehensive understanding of the effect of partisan distance therefore requires integrating evidence from both approaches.

Finally, we underscore that our critique is focused on the methodological aspects of the original study, rather than its theoretical message. We hope that this critique will stimulate constructive dialogue and further improvements in the methods used to analyse within-person effects of political attitudes on outgroup animosity.

## Appendix

### Appendix 1. Calculation of CPID and CSTR in the completely within-person model

Let WPID_ik_ represent the original party identity response (on 1-7 scale) of respondent i in wave k, and let MPID_i_ denote the respondent’s mean of WPID across all waves. The mean-centered transient changed in identity score, CPID_ik_ (BV refer to this score as Changed-Direction score). is calculated as the difference between WPID_ik_ and MPID_i_. Similarly, the mean-centered transient changed in strength score (CSTR_ik_, hereafter) is computed in three stages: First, the raw wave strength (WSTR_ik_) is computed as the absolute value of the discrepancy of WSTR_ik_ from the scale midpoint (e.g., WSTR_ik_ = ABS(CPID_ik_-4) in the case of a 7-point scale); then, the mean within-person strength (MSTR_i_) is computed; finally, the changed-strength score (CSTR_ik_) is calculated as the difference between the WSTR_ik_ and MSTR_i_. These calculations are illustrated in Table 1 in the main text.

### Appendix 2

Let equation (4) denote the level-1 model, whereby the attitude of respondent i in wave k is predicted by the centered distance in wave k. According to equation (5), the mean attitude expressed by respondent i depends on his/her long-term PID (a level-2 construct). According to equation (6) the effect of distance depends on his/her long-term PID. Using these notations, the correspondence between equation (3) and equations (4)-(6) is the following: δ0=g00, δ1=g01, δ2=g10, δ3=g11


$$\begin{array}{c} {\text{ATT}}_{\text{ik}}\;=\;{\beta \text{0}}_{\text{i}}\;+{\beta \text{1i}}_{\text{i}}*{\text{CDISTANCE}}_{\text{ik}} \end{array}$$ (4)

$$\begin{array}{c} {\beta \text{0}}_{\text{i}}\;=\;\gamma \text{00}\;+\;\gamma \text{01}{*\text{PID}}_{\text{i}} \end{array}$$ (5)

$$\begin{array}{c} {\beta \text{1}}_{\text{i}}\;=\;\gamma \text{10}\;+\;\gamma \text{11}{*\text{PID}}_{\text{i}} \end{array}$$ (6)
